# Supplementary material for: Preventing COVID-19 spread in closed facilities by regular testing of employees—An efficient intervention in long-term care facilities and prisons?
Source: PLoS One. 2021 Apr 22;16(4):e0249588. doi: 10.1371/journal.pone.0249588 (PMC8062045; doi:10.1371/journal.pone.0249588)
Supplement: S6 Fig — IFs instead of German LTCFs. Parameters for contact reduction are given in S7 Table. (PDF) [file pone.0249588.s006.pdf]

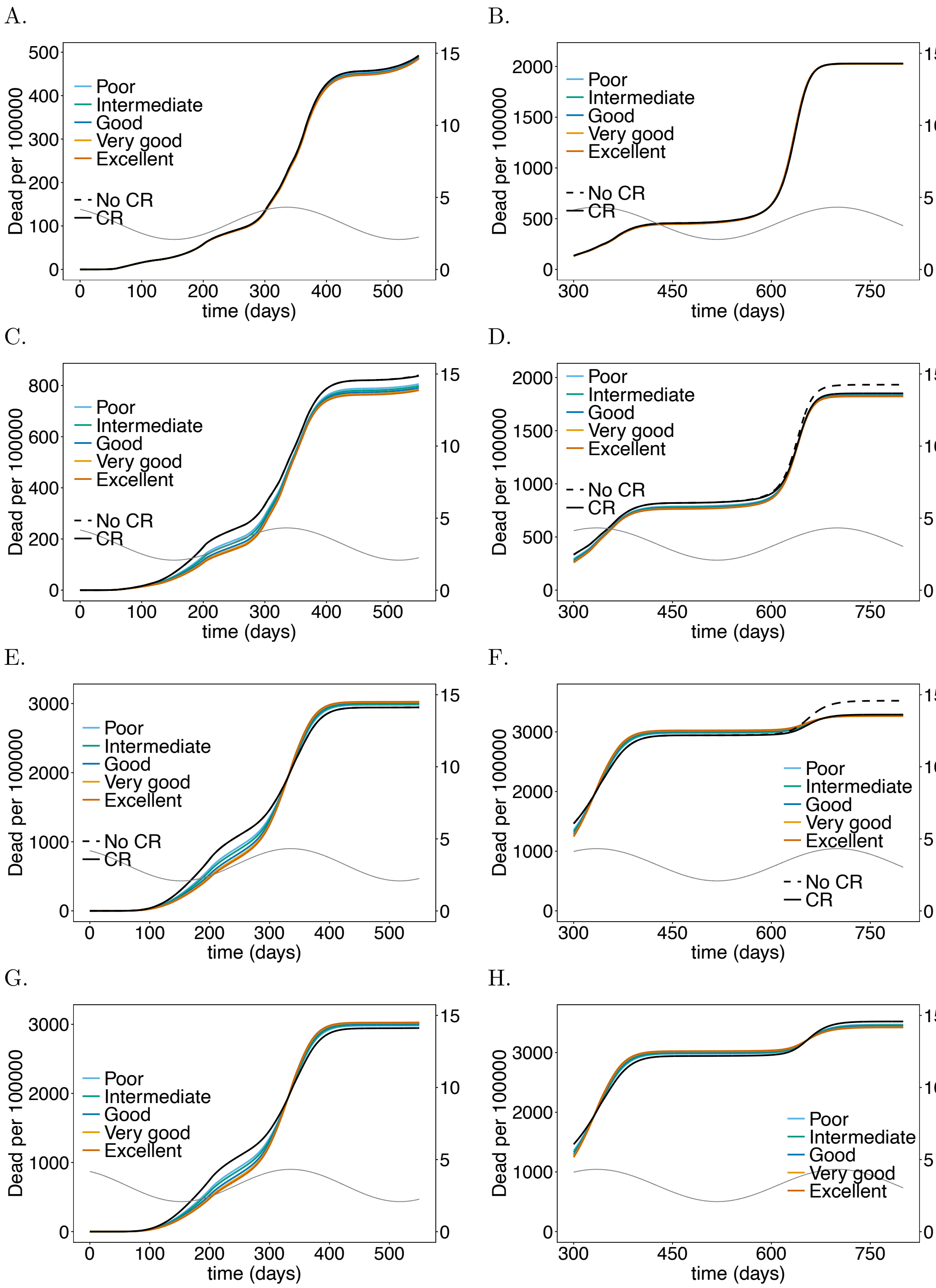

S6 Fig. Impact of sensitivity when testing IF staff on mortality:
As in Fig 7 but for U.S. IFs instead of German LTCFs. Parameters for contact reduction are given in S7 Table.
