# Supplementary material for: Preventing COVID-19 spread in closed facilities by regular testing of employees—An efficient intervention in long-term care facilities and prisons?
Source: PLoS One. 2021 Apr 22;16(4):e0249588. doi: 10.1371/journal.pone.0249588 (PMC8062045; doi:10.1371/journal.pone.0249588)
Supplement: S1 Table — (PDF) [file pone.0249588.s008.pdf]

S1 Table. (Sub-) population sizes of Germany (GER) and the USA chosen in simulations.

Parameter	Description	GER	USA
N	Total population size	83,000,000	331,000,000
$N^{(\mathrm{Ge})}$	Size of general sub-population	81,800,000	329,177,000
$N^{(\mathrm{St})}$	Number of LTCF employees	500,000	423,000
$N^{(\mathrm{Ri})}$	Size of risk group	700,000	1,400,000
