# Supplementary material for: Preventing COVID-19 spread in closed facilities by regular testing of employees—An efficient intervention in long-term care facilities and prisons?
Source: PLoS One. 2021 Apr 22;16(4):e0249588. doi: 10.1371/journal.pone.0249588 (PMC8062045; doi:10.1371/journal.pone.0249588)
Supplement: S2 Table — (PDF) [file pone.0249588.s009.pdf]

S2 Table. Summary of model parameters and their choices for numerical simulations for Germany (GER) and USA.

Parameter	Description	Value	
n_E	Number of latent phase Erlang states	16	
n_P	Number of prodromal Erlang states	16	
n_I	Number of fully infectious Erlang states	16	
n_L	Number of late infectious Erlang states	16	
D_E	Average duration of latency period	3.7 days	
D_P	Average duration of prodromal period	1 day	
D_I	Average duration of fully infectious period	5 days	
D_L	Average duration of late infectious period	5 days	
ε	Transition rate of latent states	n_E/D_E	
φ	Transition rate of prodromal states	n_P/D_P	
γ	Transition rate of fully infectious states	n_I/D_I	
δ	Transition rate of late infectious states	n_L/D_L	
$1/\alpha$	Waiting time for test results in days	1/2, 1, 2, 3, 4	
$1/\xi$	Average frequency of testing in days	1, 2, 5, 7, 14	
		GER	USA
f_{Sick}	Fraction of symptomatic (sick) infections in Ge and St	58%	58%
$f_{\text{Sick}}^{(\text{Ri})}$	Fraction of symptomatic (sick) infections in Ri	60%	43%
f_{Dead}	Fraction of sick ind. in Ge and St, who die from the disease	1.6%	4%
$f_{\text{Dead}}^{(\text{Ri})}$	Fraction of sick ind. in Ri, who die from the disease	20%	11%
f_{Iso}	Fraction of sick ind. who go to isolation	58%	48%

General sub-population (Ge), LTCF employees (St), risk group (Ri).
