# Supplementary material for: Preventing COVID-19 spread in closed facilities by regular testing of employees—An efficient intervention in long-term care facilities and prisons?
Source: PLoS One. 2021 Apr 22;16(4):e0249588. doi: 10.1371/journal.pone.0249588 (PMC8062045; doi:10.1371/journal.pone.0249588)
Supplement: S3 Table — (PDF) [file pone.0249588.s010.pdf]

S3 Table. Summary of variables describing sub-population sizes in non-infectious (sub-)states in Germany (GER) and the USA.

Name	Description	Initial value	
		GER	USA
$S^{(\mathrm{Ge})}$	No. susceptibles in Ge	81,799,800	329,176,925
$S^{(\mathrm{St})}$	No. susceptibles in St	500,000	423,000
$S^{(\mathrm{Ri})}$	No. susceptibles in Ri	700,000	1,400,000
$E_k^{(\mathrm{Ge})}$	No. infected Ge in kth latent state ($1 \leq k \leq n_E$)		0
$E_k^{(\mathrm{St},-)}$	No. infected undetected St in kth latent state ($1 \leq k \leq n_E$)		0
$E_k^{(\mathrm{St},*)}$	No. St in kth latent state, whose test results will be pos. ($1 \leq k \leq n_E$)		0
$E_k^{(\mathrm{St},+)}$	No. pos. tested St in kth latent state ($1 \leq k \leq n_E$)		0
$E_k^{(\mathrm{Ri})}$	No. Ri in kth latent state ($1 \leq k \leq n_E$)		0
$E_{\mathrm{Sum}}^{(\mathrm{Ge})}$	Total No. of Ge in latent states		0
$E_{\mathrm{Sum}}^{(\mathrm{St},-)}$	Total No. undetected infected St in latent states		0
$E_{\mathrm{Sum}}^{(\mathrm{St},*)}$	Total No. of St in latent states, whose test results will be pos.		0
$E_{\mathrm{Sum}}^{(\mathrm{St},+)}$	Total No.of pos. tested St in latent states		0
$E_{\mathrm{Sum}}^{(\mathrm{Ri})}$	Total No. of Ri in latent states		0
$R^{(\mathrm{Ge})}$	No. recovered in Ge		0
$R^{(\mathrm{St},-)}$	No. recovered in St, whose infections were undetected		0
$R^{(\mathrm{St},*)}$	No. St that recovered, before pos. test result returned		0
$R^{(\mathrm{St},+)}$	No. recovered St that were pos. tested		0
$R^{(\mathrm{Ri})}$	No. recovered in Ri		0
$D^{(\mathrm{Ge})}$	No. dead in Ge		0
$D^{(\mathrm{St},-)}$	No. dead in St, whose infections were undetected		0
$D^{(\mathrm{St},*)}$	No. St that died, before pos. test result returned		0
$D^{(\mathrm{St},+)}$	No. dead St that were pos. tested		0
$D^{(\mathrm{Ri})}$	No. dead in Ri		0

Description of variables and their initial values chosen for the simulations.
