# Supplementary material for: Preventing COVID-19 spread in closed facilities by regular testing of employees—An efficient intervention in long-term care facilities and prisons?
Source: PLoS One. 2021 Apr 22;16(4):e0249588. doi: 10.1371/journal.pone.0249588 (PMC8062045; doi:10.1371/journal.pone.0249588)
Supplement: S4 Table — (PDF) [file pone.0249588.s011.pdf]

S4 Table. Summary of variables describing sub-population sizes in infectious (sub-)states in Germany (GER) and the USA.

Name	Description	Initial value	
$P_k^{(\mathrm{Ge})}$	No. infected Ge in kth prodromal state ($1 \leq k \leq n_P$)		0
$P_k^{(\mathrm{St},-)}$	No. infected undetected St in kth prodromal state ($1 \leq k \leq n_P$)		0
$P_k^{(\mathrm{St},*)}$	No. St in kth prodromal state, whose test results will be pos. ($1 \leq k \leq n_P$)		0
$P_k^{(\mathrm{St},+)}$	No. pos. tested St in kth prodromal state ($1 \leq k \leq n_P$)		0
$P_k^{(\mathrm{Ri})}$	No. Ri in kth prodromal state ($1 \leq k \leq n_P$)		0
$P_{\mathrm{Sum}}^{(\mathrm{Ge})}$	Total No. of Ge in prodromal states		0
$P_{\mathrm{Sum}}^{(\mathrm{St},-)}$	Total No. undetected infected St in prodromal states		0
$P_{\mathrm{Sum}}^{(\mathrm{St},*)}$	Total No. of St in prodromal states, whose test results will be pos.		0
$P_{\mathrm{Sum}}^{(\mathrm{St},+)}$	Total No. of pos. tested St in prodromal states		0
$P_{\mathrm{Sum}}^{(\mathrm{Ri})}$	Total No. of Ri in prodromal states		0
		GER	USA
$I_1^{(\mathrm{Ge})}$	No. infected Ge in 1st fully infectious state	200	75
$I_k^{(\mathrm{Ge})}$	No. infected Ge in kth fully infectious state ($2 \leq k \leq n_I$)	0	0
$I_{\mathrm{Sum}}^{(\mathrm{Ge})}$	Total No. of Ge in fully infectious states	200	75
$I_k^{(\mathrm{St},-)}$	No. infected undetected St in kth fully infectious state ($1 \leq k \leq n_I$)		0
$I_k^{(\mathrm{St},*)}$	No. St in kth fully infectious state, whose test results will be pos. ($1 \leq k \leq n_I$)		0
$I_k^{(\mathrm{St},+)}$	No. pos. tested St in kth fully infectious state ($1 \leq k \leq n_I$)		0
$I_k^{(\mathrm{Ri})}$	No. Ri in kth fully infectious state ($1 \leq k \leq n_I$)		0
$I_{\mathrm{Sum}}^{(\mathrm{St},-)}$	Total No. undetected infected St in fully infectious states		0
$I_{\mathrm{Sum}}^{(\mathrm{St},*)}$	Total No. of St in fully infectious states, whose test results will be pos.		0
$I_{\mathrm{Sum}}^{(\mathrm{St},+)}$	Total No.of pos. tested St in fully infectious states		0
$I_{\mathrm{Sum}}^{(\mathrm{Ri})}$	Total No. of Ri in fully infectious states		0
$L_k^{(\mathrm{Ge})}$	No. infected Ge in kth late infectious state ($1 \leq k \leq n_L$)		0
$L_k^{(\mathrm{St},-)}$	No. infected undetected St in kth late infectious state ($1 \leq k \leq n_L$)		0
$L_k^{(\mathrm{St},*)}$	No. St in kth late infected state, whose test results will be pos. ($1 \leq k \leq n_L$)		0
$L_k^{(\mathrm{St},+)}$	No. pos. tested St in kth late infectious state ($1 \leq k \leq n_L$)		0
$L_k^{(\mathrm{Ri})}$	No. Ri in kth late infectious state ($1 \leq k \leq n_L$)		0
$L_{\mathrm{Sum}}^{(\mathrm{Ge})}$	Total No. of Ge in late infectious states		0
$L_{\mathrm{Sum}}^{(\mathrm{St},-)}$	Total No. infected undetected individuals in late infectious state in St		0
$L_{\mathrm{Sum}}^{(\mathrm{St},*)}$	Total No. of St in late infected states, whose test results will be pos.		0
$L_{\mathrm{Sum}}^{(\mathrm{St},+)}$	Total No.of pos. tested St in late infectious states		0
$L_{\mathrm{Sum}}^{(\mathrm{Ri})}$	Total No. of Ri in late infectious states		0

Description of variables and their initial values chosen for the simulations.
