# Supplementary material for: Preventing COVID-19 spread in closed facilities by regular testing of employees—An efficient intervention in long-term care facilities and prisons?
Source: PLoS One. 2021 Apr 22;16(4):e0249588. doi: 10.1371/journal.pone.0249588 (PMC8062045; doi:10.1371/journal.pone.0249588)
Supplement: S5 Table — (PDF) [file pone.0249588.s012.pdf]

S5 Table. Summary of model parameters describing the contact behavior for Germany (GER) and USA.

Name	Description	Value/Eq.	
		GER	USA
λ_{Ext}	Infections from outside of the population	45/day	50/day
$\bar{R}_0$	Annual average basic reproduction number	3.4	3.2
a	Amplitude of the seasonal fluctuation of R_0	0.43	0.35
$t_{R_{0\max}}$	Day when R_0 reaches its maximum	300	335
t_{Iso_1}	Day of beginning of case isolation measures	30	20
t_{Iso_2}	Day of end of case isolation measures	750	800
$Q_{\max}$	Maximum capacity of isolation units per 10 000	200	30
$n^{(\text{Ge})}$	Average number of daily contacts of an individual in Ge	50	60
$n^{(\text{St})}$	Average number of daily contacts of an individual in St	50	60
$n^{(\text{Ri})}$	Average number of daily contacts of an individual in Ri	30	60
x	prob. for individual in Ge to meet with individual in Ge	99.50%	99.92%
y	prob. for individual in Ge to meet with individual in St	0.30%	0.07%
u	prob. for individual in St to meet with individual in Ge	49.08%	54.47%
v	prob. for individual in the St to meet individual of the St	20.0%	20.0%
p	prob. for individual in Ri to meet individual of the Ge	38.95%	2.35%
q	prob. for individual in Ri to meet individual in St	36.81%	7.71%
c_P	Relative infectiousness in prodromal period		0.5
c_I	Relative infectiousness in fully infectious stage		1
c_L	Relative infectiousness in late infectious stage		0.5
$\beta_P(t)$	Seasonally varying effective contact rate of prodromal ind.		(23a)
$\beta_I(t)$	Seasonally varying effective contact rate of fully infectious ind.s		(23b)
$\beta_L(t)$	Seasonally varying effective contact rate of late infectious ind.		(23c)
p_{Home}	Contact reduction in home isolation		75%

Description of parameters and their initial values chosen for the simulations. Equation numbers refer to S1 Appendix
