# Supplementary material for: Preventing COVID-19 spread in closed facilities by regular testing of employees—An efficient intervention in long-term care facilities and prisons?
Source: PLoS One. 2021 Apr 22;16(4):e0249588. doi: 10.1371/journal.pone.0249588 (PMC8062045; doi:10.1371/journal.pone.0249588)
Supplement: S6 Table — (PDF) [file pone.0249588.s013.pdf]

S6 Table. Contact reduction parameters chosen for the simulations of Germany.

Parameter	Description	I_1	I_2	I_3	I_4	I_5	I_6
t_{Dist_n}-$t_{\text{Dist}_{n+1}}$	Time intervals of gen. cont. red.	40-82	82-246	246-280	280-380	380-450	450-750
	Fraction of avoided contacts in time intervals I_k between						
$p_{\text{Cont}}^{(\text{Ge,Ge})}$	Ge and Ge	0.70	0.40	0.50	0.68	0.50	0
$p_{\text{Cont}}^{(\text{Ge,St})}$	Ge and St	0.70	0.40	0.50	0.68	0.50	0
$p_{\text{Cont}}^{(\text{Ge,Ri})}$	Ge and Ri	0.60	0.20	0.40	0.50	0.40	0
$p_{\text{Cont}}^{(\text{St,St})}$	St and St	0.60	0.40	0.50	0.50	0.50	0.50
$p_{\text{Cont}}^{(\text{St,Ri})}$	St and Ri	0.35	0.20	0.20	0.35	0.35	0.35
$p_{\text{Cont}}^{(\text{Ri,Ri})}$	Ri and Ri	0.40	0.20	0.30	0.40	0.40	0.30
