# Supplementary material for: Preventing COVID-19 spread in closed facilities by regular testing of employees—An efficient intervention in long-term care facilities and prisons?
Source: PLoS One. 2021 Apr 22;16(4):e0249588. doi: 10.1371/journal.pone.0249588 (PMC8062045; doi:10.1371/journal.pone.0249588)
Supplement: Attachment — Submitted filename: COVID-19 Spread Institutional Review.pdf [file pone.0249588.s015.pdf]

PLOS One Review: COVID-19 Spread
Comments to Authors

This is a well-written and well-organized manuscript presenting a relatively clear model analyzing the impact of different intervals of testing on the number of infections and deaths among residents in long-term care facilities. I am not in expert in the underlying mathematical model used to predict these effects; I read this instead as a scholar of closed institutions, especially prisons, and focus on the theoretical framing and implications of the results. I trust other reviewers will speak more directly to the underlying mathematical model.

In terms of how these findings are presented, I have two central suggestions: one about the theoretical framing, and one about the way the mathematically modeled impacts are highlighted throought the piece.

First, while I appreciate the authors' attempt to connect the relevance of their modeling of LTCFs to incarceration facilities, as currently written, that connection feels rather under-developed and unclear, even if the underlying analogy is logical. So here are some suggestions about better developing the connection:

- I cannot tell from the way the model is described whether the authors use LTCF explicitly to mean any closed facility, nursing home or prison, or whether they are modeling details specific to LTCFs or nursing homes. Clarifying this up front will be a first step to resolving my concern about the relationship in this paper between LTCFs and other closed facilities. As I understood it, based on the methods, results, and conclusion, the model the authors ran was focused on estimating impacts explicitly for LTCFs. If this is the case, then it would be very important to know explicitly: Do the authors think the model would apply equally to carceral settings? Why or why not? They seem to suggest, towards the very end of the piece, that the main point of analogy is increasingly elderly populations, but is that it?
- If the focus of the model is, indeed, on LTCFs, then perhaps the connection to carceral facilities belongs in the discussion/implications, as an argument that the relevance of this model would likely apply equally to any other closed facility, especially carceral ones, with an explicit discussion of the similarities and differences, or limitations of the analogy.
- If, on the other hand, the authors think their model is evaluating the impact of testing on any closed facility population, that needs to be clearly explained up front, and the reasons why more thoroughly elaborated. In particular, in the introduction, the authors seem to be comparing apples to oranges across facilities: providing statistics about long-term care facilities in Germany and statistics about carceral facilities in the United States. Why these countries? Presumably the U.S. is highlighted, because their carceral infection rates are a disaster; is Germany highlighted, because their LTSC infection rates have been particularly high, or particularly low? At the very least, these examples need to be justified and contextualized. If the argument is that this model applies across these contexts, then broadening the contextualization seems especially important. Specifically, perhaps the authors could talk about relevant statistics about LTCFs in

Germany and a few other places (the UK and the US, for instance, or Europe, and other continents/regions) and then also discuss statistics about carceral settings in the *same places*.

- Finally, theoretically, the idea that LTCFs and carceral settings are very similar in terms of risks-to-residents of COVID infection, is a common one in the public health literature at this point, but the analogy is substantively and theoretically very under-developed. This article seems very well positioned to contribute to this argument theoretically and substantively. Theoretically, what are the ways that LTCFs and carceral facilities are similar? The authors seem to focus on aging populations. This seems relevant and could be more directly highlighted. But are there other points of similarity? I think there are, including other risk factors (underlying health problems, psychological isolation, etc.) shared across these populations, institutional infrastructure and policy that might not provide adequate oversight or care requirements, etc. These possible points of similarity could be developed either in the introduction (to the extent the model will be related directly to these points) or in the conclusion (to the extent the model will just be analogized to these points and have implications for other closed settings). Substantively, might the model include other factors that would make it more relevant to other closed facilities? Are there risk factors beyond age that could be included? Or might the authors highlight how the closed nature of these facilities (with the residents unable to leave) is a key piece of the analysis?

Second, the key findings of the model seem to be on page 6: “Testing LTCF staff every two weeks (14 days) leads to an almost 10-fold reduction in the number of infections and deaths compared to no testing interventions. Increasing the testing rate to 1 per week … results in a further 25% reduction …” But these findings are a bit buried. These specific mathematical estimates should be included in the abstract and highlighted as a key result of this analysis, and when presented, they could be described more clearly, especially be ensuring that each intervention (testing at 14 days, testing at 7, testing every 2) is described explicitly in terms of a percentage reduction in infections and death, rather than sometimes being described as a proportion (10-fold) and sometimes as percentage, just to avoid making the readers do math in their heads. Relatedly, the abstract could generally be more concise and focused: one sentence about the importance of testing in LTCFs in a pandemic; a more clearly stated question underlying the analysis about the impact of different intervals of testing on infection and mortality rates among confined individuals; a more precise statement of the most impactful findings. In terms of those findings, there seem to be three that are really meaningful and a bit surprising: 1. Testing every 7 days optimizes risk mitigation. 2. Low quality or slow-turn-around tests hardly affect the powerful risk reduction effects. 3. The potential offset of economic loss from this up-front investment in testing are exponential. These three take-aways should be front and center through out this piece, as they provide a clear and simple roadmap for improved policy, which should be maximally accessible if this piece is going to have an impact.
