# Supplementary material for: Preventing COVID-19 spread in closed facilities by regular testing of employees—An efficient intervention in long-term care facilities and prisons?
Source: PLoS One. 2021 Apr 22;16(4):e0249588. doi: 10.1371/journal.pone.0249588 (PMC8062045; doi:10.1371/journal.pone.0249588)
Supplement: Attachment — Submitted filename: Responses to Reviewers.pdf [file pone.0249588.s016.pdf]

Responses to Review Comments for the manuscript with number PONE-D-20-31748, entitled "*Preventing COVID-19 spread in closed facilities by regular testing of employees - an efficient intervention in long-term care facilities and prisons*"

Reviewers and suggested responses

Comments from Reviewer 1

1. **Comment**: This is a well-written and well-organized manuscript presenting a relatively clear model analyzing the impact of different intervals of testing on the number of infections and deaths among residents in long-term care facilities.

Response: We thank the reviewer for this comment. We are pleased that our manuscript's objectives have been understood and described as well organized.

2. **Comment**: I am not an expert in the underlying mathematical model used to predict these effects; I read this instead as a scholar of closed institutions, especially prisons, and focus on the theoretical framing and implications of the results.

Response: We appreciate the input from the reviewer with expertise in closed institutions. We value the reviewer's comments which we have incorporated into the revised manuscript with details given below, and believe has greatly improved the quality and readability of the revised manuscript.

3. **Comment**: I cannot tell from the way the model is described whether the authors use LTCF explicitly to mean any closed facility, nursing home or prison, or whether they are modeling details specific to LTCFs or nursing homes.

Response: This is a pertinent point raised by the reviewer. We had in mind facilities where residents, which constitute a risk group, stay for long periods and are overseen and visited by staff members who do not necessarily stay in these facilities but reside outside these facilities and therefore must mix with residents of these facilities and, as well, with a third group which we have referred to as the general population. In this way, we have in mind facilities such as retirement homes, nursing homes, and prisons. In the original description, we used the term LTCF pars pro toto. In the revised version we made the difference more explicit and added incarceration facilities in the U.S. as a further model application. Although it is the same model, the model parameters are quite different, particularly the contact behavior within prisons and LTCFs are quite different.

4. **Comment.** As I understood it, based on the methods, results, and conclusion, the model the authors ran was focused on estimating impacts explicitly for LTCFs. If this is the case, then it would be very important to know explicitly: Do the authors think the model would apply equally to carceral settings? Why or why not? They seem to suggest, towards the very end of the piece, that the main point of analogy is increasingly elderly populations, but is that it?

Response: This is an important issue raised by the reviewer. We think the model applies equally to incarceration facilities and exemplified this in the revised ms, by adjusting the model parameters to reflect the situation in US prisons. We explain differences between LTCFs and prisons in detail in the introduction, result section, and discussion of the revised ms.

5. **Comment**: If the focus of the model is, indeed, on LTCFs, then perhaps the connection to carceral facilities belongs in the discussion/implications, as an argument that the relevance of

this model would likely apply equally to any other closed facility, especially carceral ones, with an explicit discussion of the similarities and differences, or limitations of the analogy.

Response: See reply to comments 3 and 4.

6. **Comment**: If, on the other hand, the authors think their model is evaluating the impact of testing on any closed facility population, that needs to be clearly explained upfront, and the reasons why more thoroughly elaborated. In particular, in the introduction, the authors seem to be comparing apples to oranges across facilities: providing statistics about long-term care facilities in Germany and statistics about carceral facilities in the United States. Why these countries? Presumably, the U.S. is highlighted, because their carceral infection rates are a disaster; is Germany highlighted, because their LTSC infection rates have been particularly high, or particularly low? At the very least, these examples need to be justified and contextualized. If the argument is that this model applies across these contexts, then broadening the contextualization seems especially important. Specifically, perhaps the authors could talk about relevant statistics about LTCFs in Germany and a few other places (the UK and the US, for instance, or Europe, and other continents/regions) and then also discuss statistics about carceral settings in the same places.

Response: We understand the reviewer's argument and actually agree. By adding the case of U.S. prisons, we made it explicit that the model applies to incarceration facilities. In the introduction and discussion, we tried to address the points raised by the reviewer and justified why we used LTCFs in Germany and prisons in the U.S. as examples.

7. **Comment**: Finally, theoretically, the idea that LTCFs and carceral settings are very similar in terms of risks-to-residents of COVID infection, is a common one in the public health literature at this point, but the analogy is substantively and theoretically very under-developed. This article seems very well positioned to contribute to this argument theoretically and substantively.

Response: We thank the reviewer for this comment on the merits of our work. The risk-to-residents factor is very important in the setting we are investigating. In the revised ms we investigate LTCFs and prisons now separately, to further clarify similarities and differences. The general setup is equivalent: the risk group is confined to the institution and the staff is their main link to the outside world. They also share common risk factors such as pre-existing conditions. Moreover, there is an increasingly growing elderly population. However, the demography is quite different, namely, prisoners are, on average, younger than the general population with the majority of prisoners being in their twenties and thirties. In the model, this translates to a smaller fraction of symptomatic infections than in the general population (in LTCFs the fraction is larger). However, symptomatic infections are lethal with a higher probability than in the general population (because of the overrepresentation of pre-existing health conditions). Moreover, residents in LTCFs will have on average less close contacts than individuals in the general population, while prisoners have more (especially in crowded facilities like some of the prisons in Alabama). LTCFs are well equipped to nurse and isolate their residents. Contact reduction and the use of PPE are easy to realize. This is different in crowded prisons. There will also be a different propensity to comply with hygienic and contact reducing measures. Anyhow, our results in the revision - which are just based on intuitive parameter choices - highlight how difficult epidemic management is in incarceration facilities. We believe that the additional results and discussion are in line with the comments of the reviewer.

8. **Comment**: Theoretically, what are the ways that LTCFs and carceral facilities are similar? The authors seem to focus on aging populations. This seems relevant and could be more directly highlighted. But are there other points of similarity? I think there are, including other risk factors (underlying health problems, psychological isolation, etc.) shared across these populations, institutional infrastructure, and policy that might not provide adequate oversight or care requirements, etc. These possible points of similarity could be developed either in the introduction (to the extent the model will be related directly to these points) or in the conclusion (to the extent the model will just be analogized to these points and have implications for other closed settings). Substantively, might the model include other factors that would make it more relevant to other closed facilities? Are there risk factors beyond age that could be included? Or might the authors highlight how the closed nature of these facilities (with the residents unable to leave) is a key piece of the analysis?

Response: see comment 7.

Because we added incarceration facilities in the U.S. as an explicit example of the model in the ms, we needed to extend the respective parts in the introduction and discussion.

9. **Comment**: Second, the key findings of the model seem to be on page 6: “Testing LTCF staff every two weeks (14 days) leads to an almost 10-fold reduction in the number of infections and deaths compared to no testing interventions. Increasing the testing rate to 1 per week … results in a further 25% reduction …” But these findings are a bit buried. These specific mathematical estimates should be included in the abstract and highlighted as a key result of this analysis, and when presented, they could be described more clearly, especially by ensuring that each intervention (testing at 14 days, testing at 7, testing every 2) is described explicitly in terms of a percentage reduction in infections and death, rather than sometimes being described as a proportion (10-fold) and sometimes as a percentage, just to avoid making the readers do math in their heads

Response: This is a valid comment. Unfortunately, our original results were flawed, since we had a bug in the implementation of the model that we discovered during the revision (see letter to editor). Hence, the results changed substantially. We tried to keep the valid point raised by the reviewer in mind during the revision.

10. **Comment**: Relatedly, the abstract could generally be more concise and focused: one sentence about the importance of testing in LTCFs in a pandemic; a more clearly stated question underlying the analysis about the impact of different intervals of testing on infection and mortality rates among confined individuals; a more precise statement of the most impactful findings. In terms of those findings, there seem to be three that are really meaningful and a bit surprising: 1. Testing every 7 days optimizes risk mitigation. 2. Low quality or slow-turn-around tests hardly affect the powerful risk reduction effects. 3. The potential offset of economic loss from this up-front investment in testing is exponential. These three takeaways should be front and center throughout this piece, as they provide a clear and simple roadmap for improved policy, which should be maximally accessible if this piece is going to have an impact.

Response: See response to comment 9. In the revised version the abstract had to be considerably rewritten to be adjusted to the new results added and to the corrections to the original results.

Comments from Reviewer 2

1. **Comment:** The paper deals with a very important hot topic. It is well organized, clear in the rationale and appropriate in the choice of the different variables of the simulation.

Response: We appreciate and thank the reviewer for this comment.

2. **Comment:** My doubts concern the economic parts. Even if the authors said that they did a rough estimation of economic costs, it is not clear whether the values chosen are related to Germany or refer to other official sources of information (such as Diagnosis-related groups) and what these costs comprise: drug, medical staff, equipment…? Within an LTCF facility? My concern is also that authors, even if indirectly, relate flue costs with the COVID ones. I'm not sure this is correct. My suggestion is to detail this part of the analysis, better explaining their estimation.

Response: We thank the reviewer for this comment. Because the implementation of the model had a bug, the original results were flawed (see letter to the editor). In fact, testing is much less effective than claimed in the original version. This impacted also the economic considerations. We, therefore, discussed them less prominently in the abstract. From the new results, it is unclear, whether the rough estimates of the economic costs are worth the investment. In the corresponding section, we explained in more detail where the numbers are coming from. This section is mainly intended as a thought-provoking impulse, rather than a concise estimate of all direct and indirect costs. This would require more in-depth knowledge than we have. The underlying numbers for our assessment come from official reports that are now cited clearly. These official reports provide only highly aggregated numbers. Nevertheless, they give a rough idea. The section was considerably rewritten. We hope we could satisfactorily address the concerns raised by the reviewer.

Additional Editor Comments:

1. Results

a. **Comment**: line 173: Parameters for R0 should be presented with context and motivations, in particular, the assumption about the seasonal variation needs references and a discussion, since it is important for the model and for the results, given that the root causes of Covid-19 seasonality are still debated.

Response: We thank the editor for this comment. We have added a brief description of how we arrived at our estimate in the text. In fact, in the revised manuscript, we always assume seasonal variation. The reason is that by adding prisons in the U.S. as a further example and by parameterizing the model to accurately resemble the true dynamics of the infections the ms increased in length. Since the original submission of the ms, quite some time passed, and we thought that the initially presented dynamics were too simplified and no longer up to date. Since fall, the global dynamics of COVID-19 suggest seasonal fluctuations in transmissibility (due to weather conditions, UV intensity, behavior in winter vs. summer). We added some justification in the introduction. While parameterizing the model, it became immediately clear to us, that realistic dynamics never emerge without seasonal fluctuations and common-sense assumptions for contact reductions. Anyhow, we believe these assumptions are much better justified in the revised ms.

b. **Comment**: Figures showing subplots (i.e., Fig.2 and following ones), are not easy to read, being dense, and visual comparisons between plots are typically difficult. A simplification and different organization of the results could be worthwhile, to make results easier to understand. For instance, the epidemic classes (S, I, R, and D) are not independent one to the other, therefore not all of the corresponding plots are strictly needed, at least as a visual representation. For instance, plots of infections are necessary, not so to always show all the others, from Fig 2 to Fig 5.

Response: We agree with the editor. Initially, we intended to make the results as transparent as possible. However, in the revised version the focus shifted. It became necessary to show the dynamics on two time intervals: for the years 2020 and 2021. We, therefore, decided to show only plots for the number of infections and deaths. All figures (except the model flow chart), changed in the revised version. We kept the editorial comments in mind in assembling the new figures.

C. **Comment**: Also, beside visual representations, tables could be effective too, especially to ease comparisons between tests. For example, it is not immediately clear what the important information is by looking at S, R, and D panels. Similarly, it is not immediately clear how infections are changing among the four cases. For these reasons, I suggest to consider reorganizing the presentation of these results, with one that more explicitly highlights the key contributions of this work.

Response: We thank the editor for this comment. Again because of the changes we made, there was a shift in the focus of the results. We believe the revised ms substantially improved. We refrained from presenting tables because the new ms focuses on resembling roughly realistic dynamics. However, parameters were chosen only intuitively and not estimated from data - this would exceed the scope of the ms, particularly since we do not have access to appropriate data sources that would allow such estimation. We felt that quantifying the model too much but putting explicit numbers would appear too much like a fabrication of data. Anyhow, we believe the essence of the editor’s comment was addressed.
