# Supplementary material for: Preventing COVID-19 spread in closed facilities by regular testing of employees—An efficient intervention in long-term care facilities and prisons?
Source: PLoS One. 2021 Apr 22;16(4):e0249588. doi: 10.1371/journal.pone.0249588 (PMC8062045; doi:10.1371/journal.pone.0249588)
Supplement: Attachment — Submitted filename: Responses to Reviewers.pdf [file pone.0249588.s017.pdf]

Comments from Reviewer 1

Overall, I thought this paper constituted an engaged, responsive, thoughtful revision from the earlier draft. I had just a few minor thoughts about further refinement to clarify the contribution:

1. Comment: Although the authors do a much better job in this draft explaining and comparing their selection of LTCFs in Germany and IFs in the U.S., there are still a few places where these comparisons could be clarified, especially (a) in the introduction where the phrase "a similar reasoning applies" could be made more precise to introduce and specify the exact analogy between LTCFs and IFs and (b) in the IFs findings section, more explicit connections could be made to each of the LTCF sub-sections of test processing, test sensitivity, antigens, and economic analysis (this last seemed like a particularly weird absence in the IF section).

Response:

a) We added two statements in the introduction. The first one to clarify upfront that the institutions are similar in their “structure” but differ substantially in the model contact behavior. The second one makes explicit that the mathematical model applies to both types of institutions, but the parameter choices to correctly reflect the contact behavior are very different between LTCFs and IFs. We also pointed to the Discussion to indicate upfront where more information can be found.

b) We extended the result section on U.S. IFs and added the same structure as for German LTCFs to draw more parallels. We particularly added a statement on economic considerations. We refrained from putting it in numbers for two reasons. We lack the expert knowledge to find appropriate numbers for medical costs for inmates in the U.S. (particularly since costs will be very different across states). Moreover, it is clear from the simulations that testing will not be cost-efficient, and in the short run even be counterproductive. However, we added a statement indicating that the U.S. prison structure should be rethought in the context of epidemic management.

2. Comment: The economic analysis was intriguing, but it seems worth adding at least a sentence noting all the things that cannot quite be quantified (the value of a life saved, the potential costs of long-term effects of having been infected, etc.)

Response: We agree, and added an appropriate sentence at the end of the economic consideration section.

3. Comment: In the IFs findings section, I remained a bit confused, even after reading a few times, about the exact effects of testing on IF infection rates. Could the authors state the finding a bit more clearly with explicit comparisons/differences with the LTCF model (this partly relates to point 1(b)).

Response: In addition to what we added in the introduction (see response to 1b), in the section “Incarceration Facilities’’ we added section headings. In one the differences between LTCFs and IFs are described. At the beginning of this heading, we added a statement explaining that differences arise mainly due to the contact behavior and the possibilities to implement contact restrictions in LTCFs vs. IFs.

4. Comment: Are there things this model does not account for that might change the outcomes? In particular, we do not yet know the effects of variants. Might the authors devote a sentence or two to specify potential limitations, like lack of knowledge about variants or long-time viral effects, to their model?

Response: We added some lines at the end of the discussion that explains the limitations of the model.

5. Comment: Just a very minor point: the authors use "anyhow" as a transitional phrase in a few places and it feels a bit overly casual/colloquial.

Response: Thanks for pointing this out, we agree and changed the “anyhow” to less colloquial transitional phrases.

Comments from Reviewer 2

1. **Comment**: The manuscript has been revised and updated addressing reviewers’ comments and suggestions. The final version has been highly improved and can be accepted for publication.

Response: We are happy our efforts were recognized!
